# Patient-Perceived Time to Recovery After Carpal Tunnel Release

**DOI:** 10.1016/j.jhsg.2026.100973

**Published:** 2026-02-27

**Authors:** Brandon E. Earp, Dafang Zhang, Kyra A. Benavent, Jessica M. Hanley, Caleb M. Yeung, Philip E. Blazar

**Affiliations:** ∗ Division of Hand and Upper Extremity Surgery, Department of Orthopaedic Surgery, Brigham and Women’s Hospital, Boston, MA; † Harvard Medical School, Boston, MA

**Keywords:** Carpal tunnel release, Hand and upper-extremity surgery, Patient perception, Perceived recovery, Recovery

## Abstract

**Purpose:**

Carpal tunnel release (CTR) is the most commonly performed hand surgery procedure, yet there is limited literature on patients’ subjective assessment of full recovery after surgery. Our primary aim was to assess the time until patients report they have achieved full recovery.

**Methods:**

Patients who had CTR were prospectively enrolled between January 2019 and August 2020 and completed questionnaires before surgery and at standardized postoperative intervals until full recovery was reported. Study questionnaires included the Likert pain scale; the Quick Disabilities of the Arm, Shoulder and Hand Score (*Quick*DASH); and the Boston Carpal Tunnel Questionnaire (BCTQ) Symptom Severity Scale (SSS) and Functional Status Scale (FSS). Patients were also asked if they had fully recovered. We performed bivariate analyses to identify variables associated with time to patient-reported full recovery.

**Results:**

Fifty-one of 86 total patients completed the study and were included in the final cohort (59%). The mean time to patient-reported full recovery was 5.5 months. Six of 51 patients enrolled (11.8%) did not fully recover by 1 year. The mean Likert pain score, *Quick*DASH, BCTQ-SSS, and BCTQ-FSS considerably improved from before surgery to full recovery, and the mean differences in these four measures exceeded the minimal clinically important difference. Postoperative scores improved compared with preoperative scores for the four metrics above by 8, 11, 14, and 15 weeks, respectively. Higher preoperative Likert pain score, preoperative *Quick*DASH, BCTQ-SSS, and BCTQ-FSS were all associated with lack of self-reported full recovery by one year after surgery.

**Conclusions:**

Patients did not report full recovery until an average of nearly 6 months after CTR, which is substantial. The reported time to full recovery was notably longer than the time to reach minimal clinically important difference for our patient-reported outcome measures. This may be useful in preoperative discussions about recovery after surgery.

**Type of study/level of evidence:**

Prognostic II.

Carpal tunnel release (CTR) is the most commonly performed surgical procedure of the hand.^1^ The success of this procedure is often gauged with objective outcomes measures such as subsequent nerve conduction studies and physical examination findings of two-point discrimination, grip strength, and pinch strength.^2^, ^3^, ^4^, ^5^ The resolution of median nerve symptoms is frequently seen after surgery, often with variable time course for recovery of painful paresthesias, static numbness, and associated weakness.^6^

More recently, the importance of subjective outcome measures has also been recognized.^7^ Validated patient-reported outcome measures have been used to investigate the patient’s perspective, as opposed to the surgeon’s perspective. To better understand the outcomes of CTR, these patient-reported outcomes of patient perceptions of sensory, symptom, and functional recovery have been correlated with recovery in nerve conduction studies, grip and pinch strength, and two-point discrimination.^5^^,^^6^^,^^8^ Early studies of patient satisfaction showed that patients were satisfied with CTR despite variable recovery of grip strength, even in advanced cases among elderly patients.^9^^,^^10^ Patient surveys have also shown that increased severity of preoperative symptoms was associated with greater improvements in symptoms and functional status, although scores still demonstrated more symptoms than those who had milder CTR before surgery.^11^^,^^12^ Generally, patient satisfaction after carpal tunnel surgery is high.

Although there have been many validated measures assessing recovery times after carpal tunnel surgery, clinicians are often still left with limited data to answer the common questions patient ask about recovery: “How long will it take for my recovery?” or “When will I become better after surgery?” Surgeons and patients may differ in their perception of recovery. Although electrophysiological data, sensorimotor data, and some validated PROMs data are available for outcomes of CTR, there is no literature on the time until patient-reported subjective symptom or functional recovery.

This prospective cohort study sought to assess the patient-centric outcomes of perceived time to subjective recovery after undergoing isolated CTR. Patients were asked whether or not they subjectively believed fully recovered after surgery at standardized time intervals after carpal tunnel release. Our primary study objective was to quantify the time to patients’ subjective report of full recovery after CTR and to assess for factors associated with prolonged recovery.

## Materials and Methods

### Patient recruitment

This prospective study recruited patients from the clinics of three fellowship-trained orthopedic hand surgeons from January 2019 to August 2020. Institutional Review Board approval was obtained. Inclusion criteria were patients recruited from the clinics aged 18 years and older undergoing unilateral, isolated CTR. All patients underwent CTR using a previously described miniopen approach.^13^ Exclusion criteria included prior ipsilateral CTR, simultaneous bilateral CTR or those undergoing CTR concomitantly with another procedure, concomitant nonsurgically treated upper-extremity injuries or pathology, a history of multiple upper-extremity injuries (within the previous 3 months of surgery), and diabetic neuropathy as indicated in the medical record. For patients who underwent staged bilateral CTR during the study period, only the first CTR procedure was included in the study to maintain the assumption of independent observations.

Before surgery, all subjects were asked to complete a 0–10 Likert pain scale, the Quick Disabilities of the Arm, Shoulder and Hand Score (*Quick*DASH), and the Boston Carpal Tunnel Questionnaire (BCTQ) Symptom Severity Scale (SSS) and Functional Status Scale (FSS).^7^^,^^8^^,^^14^ Subjects completed questionnaires via Research Electronic Data Capture surveys, which were collected via telephone by research staff or in-person by the patient on the day of surgery.^15^ The time from preoperative survey completion to surgery averaged 8.1 days (range 0–86 days).

After surgery, patients completed a Likert pain scale, *Quick*DASH, and BCTQ questionnaire at 4 weeks, 6 weeks, 3 months, 6 months, 9 months, and 12 months or until the patient-reported feeling fully recovered.^7^^,^^8^^,^^14^ Patients were also asked if they believed fully recovered at every time point with response options being “Yes,” “Somewhat,” or “No.” The authors intentionally provided no guidance to patients about the definition of “full recovery” in order to capture the patients’ subjective experience. Research Electronic Data Capture electronic contacts were used to administer the surveys by email or patients were contacted by telephone if email contact failed. Upon patients’ report of achieving full recovery, the patients were deemed to have completed the study and no further questionnaires were distributed.

### Primary response variable and explanatory variables

The time until patient-reported full recovery was our primary response variable. Other variables included (1) patient-reported full recovery by 6 months after surgery and (2) patient-reported full recovery at or before 1 year after surgery. Explanatory variables included sex, age, employment status, laterality of CTR, current tobacco or alcohol use, present diagnosis of anxiety or depression as indicated in the medical record, Worker’s Compensation insurance, electrodiagnostic status (EDS), preoperative patient-reported outcome measures (PROMs; Likert pain score, *Quick*DASH, BCTQ-SSS, and BCTQ-FSS). EDS severity was classified as mild, moderate, or severe using the Werner and Andary criteria.^16^

### Statistical analysis

Descriptive statistics were calculated. We performed a bivariate analysis assessing variables associated with time to patient-reported full recovery. Time to recovery was analyzed as a parametric continuous variable, as skewness of this variable was –0.11. Associations between time to recovery and continuous explanatory variables were assessed with linear regression. Associations between time to recovery and dichotomous explanatory variables were assessed with Student *t* test, and associations between time to recovery and nondichotomous categorical explanatory variables were assessed with analysis of variance. Significance criterion of α = 0.05 and power criterion of (1-β) = 0.80 was used for statistical tests. In accordance with prior literature on patient-reported time to recovery after trigger digit release, assuming a mean time to full recovery of 6 months with a standard deviation of 2 months, assuming 1:1 distribution among groups, a sample size of 32 patients was expected to provide 80% power to detect a notable difference in full recovery time of 2 months.^17^

A difference of 0.9 on the Likert pain scale was used as the minimal clinically important difference (MCID), and a difference of 14 on the *Quick*DASH was used as the MCID.^17^, ^18^, ^19^ A relative change of 46% was used as the MCID for BCTQ-SSS, and a relative change of 28% was used as the MCID for BCTQ-FSS.^18^^,^^19^

## Results

Eighty-six study subjects were enrolled. Thirty-five patients were excluded because of incomplete follow-up, of which 23 patients did not complete any questionnaires after surgery, and 12 did not complete any questionnaire after the 2-week time point. The final cohort included 51 subjects. The average age of the cohort was 65.9 (SD, 13.1), and 32 patients were women (Table 1). Preoperative Likert pain score, *Quick*DASH, BCTQ-SSS, and BCTQ-FSS are presented in the Table 2. At one year, 6 of 51 subjects did not report full recovery. The average time to patient-reported full recovery after CTR of the remaining 45 subjects was 5.5 months (SD, 2.6). The final Likert pain score, *Quick*DASH, BCTQ-SSS, and BCTQ-FSS of the patients who reported full recovery are also shown in the Table 2. One patient experienced a postoperative complication (incision site infection) and was treated with antibiotics. No patient underwent reoperation.

**Table 1:** Patient Demographics and Clinical Characteristics

Variable	Outcome
Age [mean (SD)]	65.9 (13.1)
Sex [n (%)]	
Female	32 (62.7)
Male	19 (37.3)
Depression [n (%)]	14 (27.5)
Anxiety [n (%)]	14 (27.5)
Laterality [n (%)]	
Right	33 (65)
Left	18 (35)
Tobacco status [n (%)]	
Never	29 (56.8)
Former	21 (41.2)
Current	1 (1.9)
Alcohol use status [n (%)]	
Never	21 (41.2)
Moderate	30 (58.8)
Worker’s compensation [n (%)]	0 (0)
Employment status∗ [n (%)]	
Employed	26 (50.9)
Retired	22 (43.1)
Unemployed	2 (3.9)
EMG result [n (%)]	
Mild	8 (19)
Moderate	17 (40.5)
Severe	17 (40.5)

∗ Missing employment status for 1 patient.

**Table 2:** Patient-Reported Outcome Measure Scores Before Surgery and at Perceived Full Recovery After Surgery (Median 5.5 Months)

Outcome Measure	Preoperative Score [Mean (SD)]	Postoperative Score at Perceived Full Recovery [Mean (SD)]
Likert Pain Score	4.4 (3)	1.1 (1.1)
BCTQ-SS	2.8 (0.8)	1.4 (0.4)
BCTQ-FS	2.3 (0.8)	1.3 (0.4)
*Quick*DASH	61.6 (19.7)	33.5 (12.2)

The mean Likert pain score, *Quick*DASH, BCTQ-SSS, and BCTQ-FSS considerably improved from before surgery to full recovery (*P* < .05), and the mean differences in these four measures exceeded the MCID. Postoperative Likert pain scores improved compared with the preoperative pain score by the MCID at an average of 8 weeks. The postoperative *Quick*DASH score improved compared with the preoperative score by the MCID at an average of 11 weeks. The postoperative BCTQ-SSS improved compared with the preoperative score by the MCID at an average of 14 weeks. The postoperative BCTQ-FSS improved compared with the preoperative score by the MCID at an average of 15 weeks.

No variables were notably associated with time to patient-reported full recovery in bivariate analyses (Table 3). No secondary response variables were considerably associated with patient-reported full recovery by 6 months after surgery in bivariate analyses. Higher preoperative scores on the Likert pain scale, *Quick*DASH, BCTQ-SSS, and BCTQ-FSS were significantly associated with lack of full recovery by one year after surgery (*P* < .05; Table 4). EDS severity was not associated with patient-reported time to recovery or achieving full recovery at 6 months or 1 year.

**Table 3:** Bivariate Analysis of Variables Associated With Time to Full Recovery After CTR

Patient Characteristic	Time to Full Recovery	
	β Regression Coefficient (SE)	*P* Value
Age	0.01 (0.03)	.5
Preoperative Likert pain score	0.01 (0.19)	.9
Preoperative *Quick*DASH	–0.001 (0.04)	.9
Preoperative BCTQ-SSS	–0.69 (0.77)	.3
Preoperative BCTQ-FSS	0.19 (0.97)	.8
	**Mean (SD)**	***P* Value**
Sex		
Female	5.2 (2.5)	.3
Male	5.9 (2.5)	
Anxiety		.07
Yes	4.3 (2.5)	
No	5.8 (2.4)	
Depression		.2
Yes	4.6 (3.1)	
No	5.7 (2.3)	
Current tobacco use		-
Yes	-	
No	5.5 (2.5)	
Current alcohol use		.3
Yes	5.1 (2.5)	
No	5.9 (2.5)	
Laterality		
Right	5.5 (2.7)	.9
Left	5.4 (2.4)	
Employment status		
Unemployed	6 (-)	
Employed	5.1 (2.3)	0.6
Retired	5.8 (2.8)	
EMG severity		
Mild	5.6 (1.6)	
Moderate	4.7 (2.6)	0.1
Severe	6.7 (2.9)	

FSS, Functional Severity Scale.

**Table 4:** Bivariate Analysis of Variables Associated With Full Recovery by 6 and 12 Months After Surgery After CTR

Patient Characteristic	6 Mo After Surgery			12 Mo Postoperative		
	Recovered	Not Recovered	*P* Value	Recovered	Not Recovered	*P* Value
	Mean (SD)	Mean (SD)		Mean (SD)	Mean (SD)	
Age	64 (11)	68 (16)	.3	66 (12)	63 (16)	.6
Preoperative Likert pain score	4 (2)	4 (3)	.5	4 (2.8)	7 (2.7)	<.05
Preoperative *Quick*DASH	59 (19)	65 (21)	.3	59 (18)	81 (21)	<.05
Preoperative BCTQ-SSS	2.9 (0.8)	2.8 (0.73)	.7	2.7 (0.7)	3.5 (0.8)	<.05
Preoperative BCTQ-FSS	2.1 (0.8)	2.5 (0.8)	.1	2.1 (0.7)	3.2 (0.7)	<.05
	**n (%)**	**n (%)**	***P* Value**	**n (%)**	**n (%)**	***P* Value**
Sex						
Female	24 (75)	8 (25)	.5	28 (87)	4 (13)	.9
Male	12 (63)	7 (37)		17 (89)	2 (11)	
Laterality						
Right	22 (66)	11 (34)	.5	28 (84)	5 (16)	.4
Left	14 (77)	4 (23)		17 (94)	1 (6)	
Anxiety			.6			.3
Yes	11 (78)	3 (21)		11 (78)	3 (22)	
No	25 (67)	12 (32)		34 (91)	3 (9)	
Depression			.9			.3
Yes	10 (71)	4 (29)		11 (78)	3 (22)	
No	26 (70)	11 (30)		34 (91)	3 (9)	
Current tobacco use			.9			
Yes	1 (100)	0 (0)		0 (0)	1 (100)	
No	35 (70)	15 (30)		45 (90)	5 (10)	.1
Current alcohol use			.8			
Yes	22 (73)	8 (26)		26 (86)	4 (14)	
No	14 (66)	7 (34)		19 (90)	2 (10)	.9
Employment status						
Unemployed	1 (50)	1 (50)		1 (50)	1 (50)	
Employed	22 (84)	4 (15)	.08	23 (88)	3 (12)	.3
Retired	13 (59)	9 (41)		20 (90)	2 (10)	
EMG Severity						
Mild	6 (85)	1 (15)	.2	7 (88)	1 (12)	.9
Moderate	13 (81)	3 (19)		16 (94)	1 (6)	
Severe	8 (53)	7 (47)		15 (88)	2 (12)	

FSS, Functional Severity Scale.

## Discussion

CTR is the most commonly performed surgical procedure in the hand and produces reliably successful outcomes.^1^, ^2^, ^3^ These measures of success most often include physical examination findings such as two-point discrimination, grip strength, and pinch strength but may also include objective outcomes such as postoperative nerve conduction studies.^2^, ^3^, ^4^, ^5^, ^6^ Indeed, the resolution of median nerve symptoms is frequently seen after surgery. Recovery of two-point discrimination has been reported as early as within 10 days.^6^

The importance of subjective patient-reported outcomes using validated measures has been increasingly recognized as part of a patient-centric approach to health care, focusing on the patient experience. These subjective outcomes of patient perceptions of sensory, symptom, and functional recovery have been correlated with the objective postoperative improvements in nerve conduction studies, grip and pinch strength, and two-point discrimination.^5^^,^^6^^,^^8^ Other studies have demonstrated that patients were satisfied with CTR despite variable objective recovery, even in severe cases among older patients.^9^, ^10^, ^11^ Surveys have also correlated more severe preoperative symptoms with greater improvements in symptoms and functional status after CTR, although ultimately the postoperative scores for those with severe CTS were still worse than for those who had milder CTS before surgery.^12^ Patient satisfaction after carpal tunnel surgery is generally high.

Outcomes and recovery after CTR are multifactorial and different for each patient. For example, there is controversy in the literature regarding the impact of age on outcomes after CTR. Several studies have indicated that PROMs are independent of age.^9^^,^^20^ Others have noted worse outcomes in older patients.^21^, ^22^, ^23^, ^24^ Hobby et al^24^ described the relationship of age and sex on carpal tunnel symptoms and outcomes after surgery. Their article demonstrated that although most patients over the age of 70 reported an improvement in symptoms and function, they were less satisfied with their treatment than younger patients. They hypothesized that this was because of variable presentation of CTS between different age groups.

**Figure 1:**
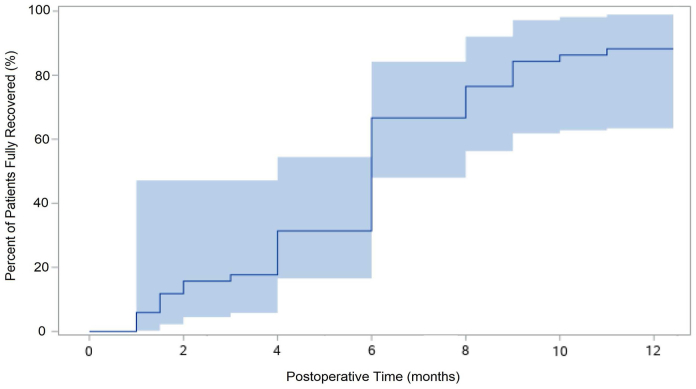
Cumulative incidence curve depicting the percent of fully recovered patients over time. The shaded area depicts the 95% Hall–Wellner band, a confidence band for the survival function accounting for uncertainty across all time points.

The mean time that patients believed fully recovered in our study was just under 6 months (Fig. 1). This study was undertaken because of a growing awareness of the differences in patient and surgeon perceptions of the time to achieve “recovery” after CTR. In addition, nearly 12% of subjects did not report full recovery by 12 months after CTR. Higher PROM values before surgery were associated with failure to achieve full recovery after CTR. These findings suggest the subjective patient experience of recovery may be influenced by variables other than the severity of the preoperative surgical diagnosis.

Interestingly, postoperative PROMs demonstrated improvements equal to or greater than the MCID well before patients reported full recovery (8–15 weeks vs 5.5 months). This indicates that current available and commonly used patient-reported outcome measures do not fully capture patients’ experience of recovery after CTR. Additions to current PROMs or perhaps new PROMs may be necessary to capture this component of the patient experience.

Limited literature is available assessing patients’ experience of recovery after CTR. Recovery after surgical treatment for trigger digit release and after open lower limb fractures has been assessed, with patient-reported recovery time that differs between physicians and patients, and paralleled improvements in functional patient-reported outcomes.^17^^,^^25^ As we noted in this current study, a sizable subgroup of patients did not achieve full patient-reported recovery by the end of the study.

This study has several limitations. First, there are not any validated instruments available which are applicable to hand surgery which measure the subjective patient experience of full recovery. There is likely variability in how patients assess this for themselves and therefore our results may not translate to all populations. Second, this study includes a relatively small number of patients with follow-up of 1 year; larger numbers or longer follow-up might influence outcomes. For example, a greater proportion of patients with severe preoperative electrodiagnostic disease did not recover after CTR. These findings did not reach statistical significance because of possible type 2 error and warrant further investigation. We encourage future studies to elucidate how the recovery trajectory may differ among various populations and subgroups after this common hand surgery. Third, our study had a high number of patients passively withdrawing from the study before or after their first postoperative visit: 35 patients were excluded for failing to complete postoperative questionnaires after CTR or after the 2-week postoperative point. If this subgroup of patients experienced full recovery more quickly, then our findings may overstate time to recovery after CTR. Fourth, our study was designed for one year of postoperative follow-up, and consequently, we are not able to comment on when or whether the remaining “unrecovered” six patients subsequently would have reported full recovery. Although EDS severity did not correlate with time to recovery in this study, it is possible that with a larger study, an effect would be seen. Fifth, we assessed outcome variables at a limited number of specific times, which may have overestimated the time of recovery if patients recovered prior to the next assessment point. Investigating these issues at more frequent time points might lead to a slightly different perceived length of time to recovery. Sixth, although we included anxiety and depression diagnoses as variables, we did not use a pain catastrophizing scale or other psychosocial instruments in this study. Future studies could assess the impact of these factors on recovery. Finally, there are potentially unstudied confounders to postoperative recovery, which also may affect a patient’s experience of full recovery.

This study demonstrated that patients report not feeling fully recovered after CTR for a mean of nearly 6 months, which is notably longer than previously published proxies for recovery such as return to work and scar tenderness, which are on the order of 1 month to 3 months, respectively.^26^ Interestingly, patients demonstrated notable improvements meeting the MCID of multiple validated outcome measures months before patients reported experiencing full recovery. This indicates currently unmeasured factors affecting patient-reported subjective recovery. Our study supports the well-established literature that CTR is a successful procedure, but adds new direction on the more prolonged subjective recovery as part of the patient experience following this surgery. These findings can help inform the preoperative discussion about reasonable expectations following CTR surgery and better inform the shared decision-making process.

## Conflicts of Interest

No benefits in any form have been received or will be received related directly to this article.
